# Salvage Radiotherapy for Macroscopic Local Recurrence Following Radical Prostatectomy

**DOI:** 10.3389/fonc.2021.669261

**Published:** 2021-04-15

**Authors:** Hind Zaine, Benjamin Vandendorpe, Benoit Bataille, Thomas Lacornerie, Jennifer Wallet, Xavier Mirabel, Eric Lartigau, David Pasquier

**Affiliations:** ^1^ Academic Department of Radiation Oncology, Centre Oscar Lambret, Lille, France; ^2^ Department of Medical Physics, Centre O. Lambret, Lille, France; ^3^ Department of Biostatistics, Centre O. Lambret, Lille, France; ^4^ CRIStAL (Centre de Recherche en Informatique, Signal et Automatique de Lille [Research center in Computer Science, Signal and Automatic Control of Lille] UMR (Unité Mixte de Recherche [joint research center]) 9189, Lille University, Lille, France

**Keywords:** prostate cancer, macroscopic recurrence, salvage radiotherapy, boost, post-therapeutic toxicity

## Abstract

**Introduction:**

Salvage radiotherapy is the only curative treatment for biochemical progression after radical prostatectomy. Macroscopic recurrence may be found in the prostatic bed. The purpose of our study is to evaluate the effectiveness of salvage radiotherapy of the prostate bed with a boost to the area of the macroscopic recurrence.

**Material and Methods:**

From January 2005 to January 2020, 89 patients with macroscopic recurrence in the prostatectomy bed were treated with salvage radiotherapy +/- hormone therapy. The average PSA level prior to radiotherapy was 1.1 ng/mL (SD: 1.6). At the time of biochemical progression, 96% of the patients had a MRI that revealed the macroscopic recurrence, and 58% had an additional choline PET scan. 67.4% of the patients got a boost to the macroscopic nodule, while 32.5% of the patients only underwent radiotherapy of the prostate bed without a boost. The median total dose of radiotherapy was 70 Gy (Min.: 60 – Max.: 74). The most commonly-used regimen was radiotherapy of the prostatectomy bed with a concomitant boost. 48% of the patients were concomitantly treated with hormone therapy.

**Results:**

After a median follow-up of 53.7 months, 77 patients were alive and 12 had died, of which 4 following metastatic progression. The 5-year and 8-year survival rates (CI95%) are, respectively, 90.2% (78.9-95.6%) and 69.8% (46.4-84.4%). The 5-year biochemical progression-free survival rate (CI95%) is 50.8% (36.7-63.3). Metastatic recurrence occurred in 11.2% of the patients. We did not find any statistically significant impact from the various known prognostic factors for biochemical progression-free survival. No toxicity with a grade of > or = to 3 was found.

**Conclusions:**

Our series is one of the largest published to date. Salvage radiotherapy has its place in the management of patients with biochemical progression with local recurrence in the prostate bed, with an acceptable toxicity profile. The interest of the boost is to be evaluated in prospective trials.

## Highlights

- Salvage radiotherapy of the prostatectomy bed usually remains the only curative treatment for recurrence after prostatectomy for prostate cancer.
- Radiation oncologists are increasingly faced with macroscopic disease detected in the prostatectomy bed.
- There is no consensus and so there is considerable variability in the management of this category of patients.
- We present one of the largest series of patients with macroscopic recurrence treated by radiotherapy to date.
- Five years after radiotherapy, around half of the patients presented with a new relapse.
- A boost to the recurrence did not influence relapse free survival and toxicity was low.
- The interest of the boost is evaluated in prospective trials currently.

## Introduction

Radical prostatectomy is an effective curative therapy and is widely used for localized prostate cancers. However, 15 to 40% of operated patients develop biochemical progression within five years after surgery (1, 2).

Salvage radiotherapy of the prostatectomy bed usually remains the only curative therapy indicated from a PSA level > 0.2 ng/ml. The effectiveness of this therapy depends on the PSA level, and some studies specify that the treatment is more effective when the pre-treatment PSA is less than 0.5 ng/mL (3, 4).

The benefits of additional hormone therapy vary depending on the pathological characteristics and make it possible to prolong metastasis-free survival (5, 6).

With the progress achieved in imaging (prostate MRIs, choline PET scans) and more recently PSMA PET CT, which is sensitive at PSA levels of less than 1 ng/mL or even 0.5 ng/mL (7, 8), radiation oncologists are increasingly faced with occurrences of biochemical progression with macroscopic disease found in the prostatectomy bed.

A 66 Gy dose, which is commonly used to treat biochemical progression, may be insufficient in cases of macroscopic recurrence, and increasing the doses applied to these recurrences is common.

To date, there is no consensus with regard to the application of a boost (target volumes, techniques, total dose, fractionation, etc.) and so there is considerable variability in the management of this category of patients with macroscopic recurrence.

The purpose of this analysis is to study this category of patients with macroscopic recurrence in the prostatectomy bed; to evaluate, retrospectively, the effectiveness of salvage radiotherapy with boost to the recurrence; and lastly, to define what place hormone therapy has in this situation.

## Material and Methods

After having obtained the patients consent to the use of their data, we conducted a retrospective study of the patients treated by radiotherapy of the prostate bed at the Centre Oscar Lambret between January 2005 and January 2020 and who had an identified macroscopic recurrence. All patients treated consecutively were included.

A macroscopic local recurrence was defined by a relapse in prostatectomy bed visible on MRI and/or CT scan and/or choline PET and/or accessible to clinical examination by digital rectal examination.

89 patients were included; their average age when diagnosed was 61.3 years (SD = 5.7). The average pre-operation PSA level was 9.4 mg/mL (SD = 4.9). The surgical stage according to AJCC TNM, 8th Edition, was, for 36%, stage pT3a; for 20%, pT3b; and for 20%, pT2c. Lymph node dissection was performed in 67% of the patients and came back negative (pN0) for all of them. The Gleason score was 7 in 77% of the cases, less than 7 in 14% of the cases, and greater than 7 in 8.9% of the cases. The resection margin was R0 in 51% of the patients. The post-operation PSA nadir could not be measured in 87% of the patients. 53% of the patients developed post-operation urinary complications, mostly grade 1 (40% of the operated patients) (**Table 1**).

**Table 1:** Patient characteristics.

Radical prostatectomy	Population N = 89	
**pTNM: T (MD = 5)**		
pT2a	7	8%
pT2b	13	15%
pT2c	17	20%
pT3a	30	36%
pT3b	17	20%
**pTNM: N (MD = 3)**		
N0	58	67%
Nx (no lymph node dissection)	28	33%
**Gleason Score (MD = 3)**		
Gleason <= 7	78	91%
Gleason >= 8	8	9%
**Resection margin (MD = 5)**		
R0	43	51%
R1	41	49%
**Post-op PSA**		
Not measurable	77	87%
Measurable	12	13%
**Postoperative urinary toxicities (MD=2)**		
Grade 1	35	40.2%
Grade 2	11	12.6%

MD, missing data.

The median time to post-prostatectomy biochemical progression was 2.3 years (Min.: 0.1-Max.: 18.9)

Multiparameter magnetic resonance imaging was performed in all except four patients who had a prostate bed nodule that was palpable on digital rectal examination and visible in the pelvic computed tomography. The median size of the prostate bed nodule was 9.5 mm (Min.: 2-Max.: 35). The recurrence was most often localized in the perianastomotic position (38.8%). 58% of the patients had had a choline PET scan, which showed hyperfixation at the macroscopic nodule in 21% of cases. Pelvic lymph node recurrence was found in 6% of the patients. A biopsy of the prostate bed nodule was performed in 20% of the patients and was positive for 10% of the patients (**Table 2**).

**Table 2:** Characteristics of the macroscopic recurrence following radical prostatectomy.

Characteristics	Population N = 89	
**Location of the macroscopic recurrence on the MRI (MD=4)**
Perianastomotic	33	38.8%
Periurethral	5	5.8%
Residual SV or SV bed	10	11.7%
Other	37	43.5%
**Size of the macroscopic recurrence on the MRI, in mm (MD = 13)**		
Median - (Range)	9.5	(2-35)
Mean – SD	11.3	6.6
**PET scan**		
Done	52	58%
*Non-hypermetabolic recurrence*	33	37%
*Hypermetabolic recurrence*	19	21%
**Biopsy of the recurrence**		
Done	18	20%
Negative	9	10%
Positive	9	10%

MD, Missing data; LR, Local recurrence.

15% of the patients had been treated prior to the salvage radiotherapy: 11% had had hormone therapy, 2% had had chemotherapy in combination with hormone therapy (Rising PSA clinical trial) and 2% had had stereotactic pelvic lymph node radiotherapy in combination with hormone therapy.

The average PSA level prior to starting radiotherapy was 1.1 ng/mL (SD = 1.6). The average PSA doubling time was 10.7 months (SD = 11.7).

The radiotherapy techniques used were intensity-modulated radiotherapy in 77.5% of the patients and the three-dimensional technique in 22.4% of them.

The median total dose of radiotherapy was 70 Gy (Min.: 60 – Max.: 74); the median dose applied to the prostate bed was 66Gy (Min.:50– Max.:66.6). The median boost fractionation was 2.1 Gy/fraction (Min.: 1.8 – Max.: 6). The median duration of the radiotherapy was 48 days.

**Figure 1:**
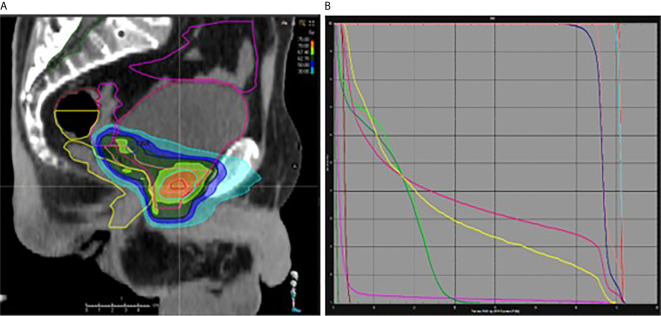
**(A, B)** Intensity Modulated Radiation Therapy of the prostatectomy bed (66 Gy) with a concomitant boost to the macroscopic recurrence (70.95 Gy). Sagittal view **(A)** and dose volume histogram **(B)**. Pink and blue: prostatic bed and macroscopic recurrence CTV and PTV; yellow: rectum; red: bladder; green: femoral heads.

The most commonly-used regimen was radiotherapy of the prostatectomy bed with a concomitant supplementary dose (boost) to the macroscopic recurrence (**Figures 1A, B**). 67.4% of the patients treated by salvage radiotherapy received a boost to the macroscopic nodule, applied concomitantly with intensity modulation in 56.66% of them, and sequentially in 43.33% of them. 32.6% of the patients had radiotherapy of the prostate bed alone with no boost. 25% of patents underwent pelvic lymph node irradiation (**Table 3**).

**Table 3:** Radiotherapy treatment methods.

	Population treated				
Total RT dose, in Gy	Median - (Range)	70		(60; 74)	
	Mean – SD	68.8		(2.5)	
RT techniques applied to the prostate bed	IMRT: 77.5%				
	3D: 22.4%				
Boost RT techniques	IMRT: 86.66%				
	3D: 6.66%				
	Stereotactic: 6.66%				
Regimens and target volumes	Prostate bed + boost: 67.4%		Concomitant boost: 56.66%		Boost fractionation: Gy Median - (Range) 2.1 - (1.8; 6) Mean – SD: 2.3 – 0.9
			Sequential boost: 43.33%		
	Prostate bed, no boost: 32.6%				
	Pelvic lymph node irradiation: 25%				

RT, Radiotherapy; Boost, supplementary dose; IMRT, intensity-modulated radiotherapy; 3D, three-dimensional radiotherapy; Fr, fractionation.

We compared the two groups (with boost and without boost) in terms of median follow-up, baseline PSA, size of the macroscopic recurrence, the use or not of ADT and the choice of radiotherapy technique. The two groups were well balanced except for the technique and follow up. In the boost group, IMRT was used more often (90% vs 51.7%, p< 0.001) and the median follow up was shorter: 45 months (40-54 months) vs 61.4 months (51-72 months), p = 0.03.

We recorded the acute toxicities (during the radiotherapy and within three months post-treatment) and the delayed ones (more than three months after the end of the treatment). These toxicities were graded on the CTCAE scale, version 4.03.

48% of the patients had hormone therapy in combination with the radiotherapy, most often for a short period of time (6 months) (45%). Post-radiotherapy patient follow-up was carried out alternatively with the urologists, on a quarterly basis in the first year and then every six months, with a PSA screening performed prior to each consultation.

Remission is defined by a post-radiotherapy PSA nadir level less than the pre-radiotherapy PSA level. There being no consensual definition about biochemical progression after salvage radiotherapy, we opted for two definitions in our study: PSA > 0.2 ng/mL (definition 1) and post-radiotherapy PSA > PSA nadir + 0.5 ng/mL (definition 2). The latter definition was used in a recent retrospective study (9). Rising PSA was confirmed by two screenings one month apart.

Clinical recurrence is defined by the detection of a local, pelvic lymph node, recurrence or distant metastatic recurrence on imaging studies. A second imaging may be performed at the doctor’s discretion.

### Statistical Analysis

Biochemical progression-free survival (main criterion) with no metastatic or local recurrence and overall survival (secondary criteria) were estimated using the Kaplan-Meier method from the radiotherapy start date.

The prognostic value of the PSA level at the start of the treatment and the prognostic value of the boost with regard to biochemical progression-free survival were assessed using Cox regression models. The threshold of significance was set at 5%.

The software used was Stata v15.0 (StataCorp. 2009. Stata Statistical Software: Release 11. College Station, TX: StataCorp LP).

## Results

Post-radiotherapy remission was achieved in 93% of the patients, 79% of whom had a PSA nadir below 0.1 ng/mL.

**Figure 2:**
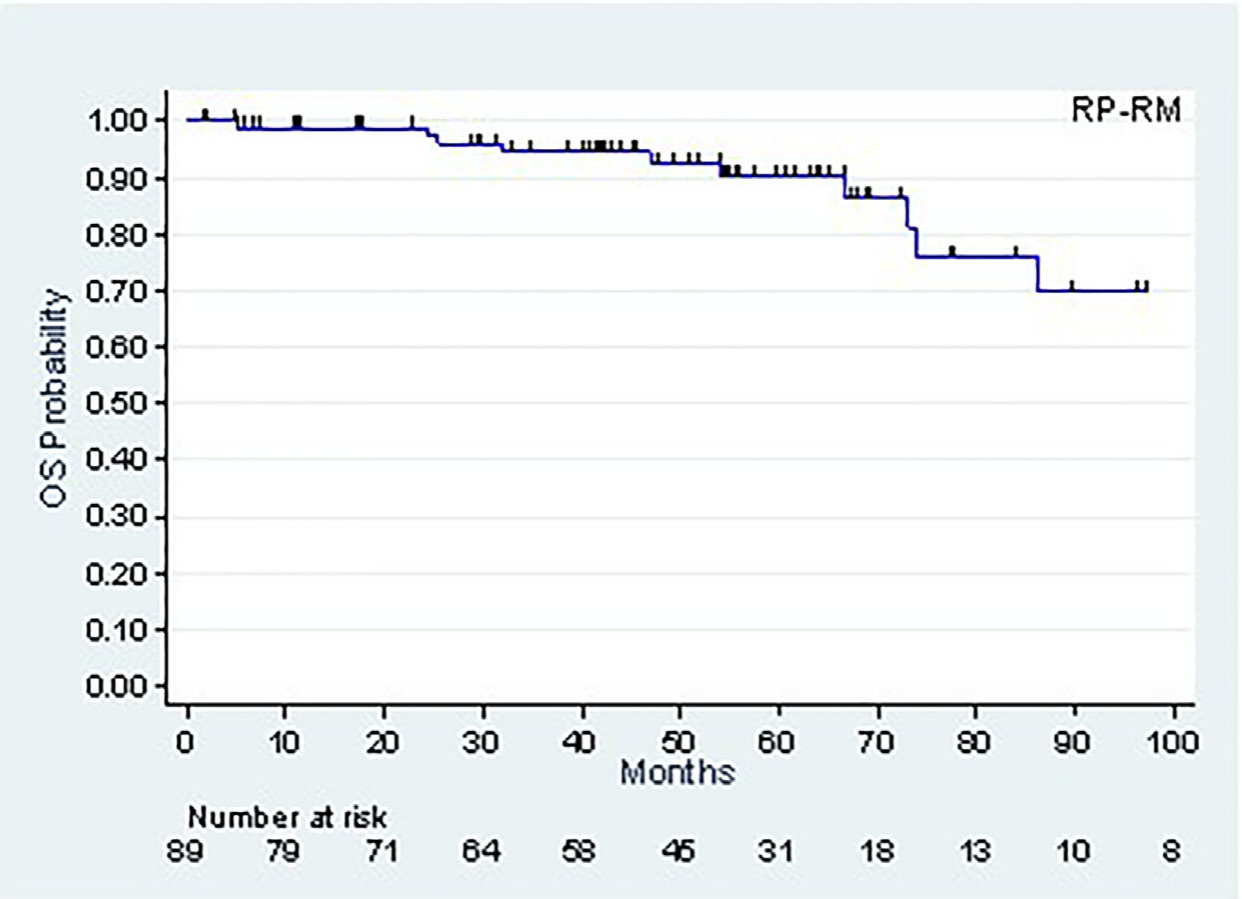
Overall survival.

The patients’ follow-up, calculated using the Kaplan-Meier method, was 53.7 months (42.8-59.4 months). As of this follow-up, 77 patients were alive and 12 had died, of which 4 following metastatic progression. The 5-year (CI95%) and 8-year survival rates were, respectively, 90.2% (78.9-95.6%) and 69.8% (46.4-84.4%) (**Figure 2**).

**Figure 3:**
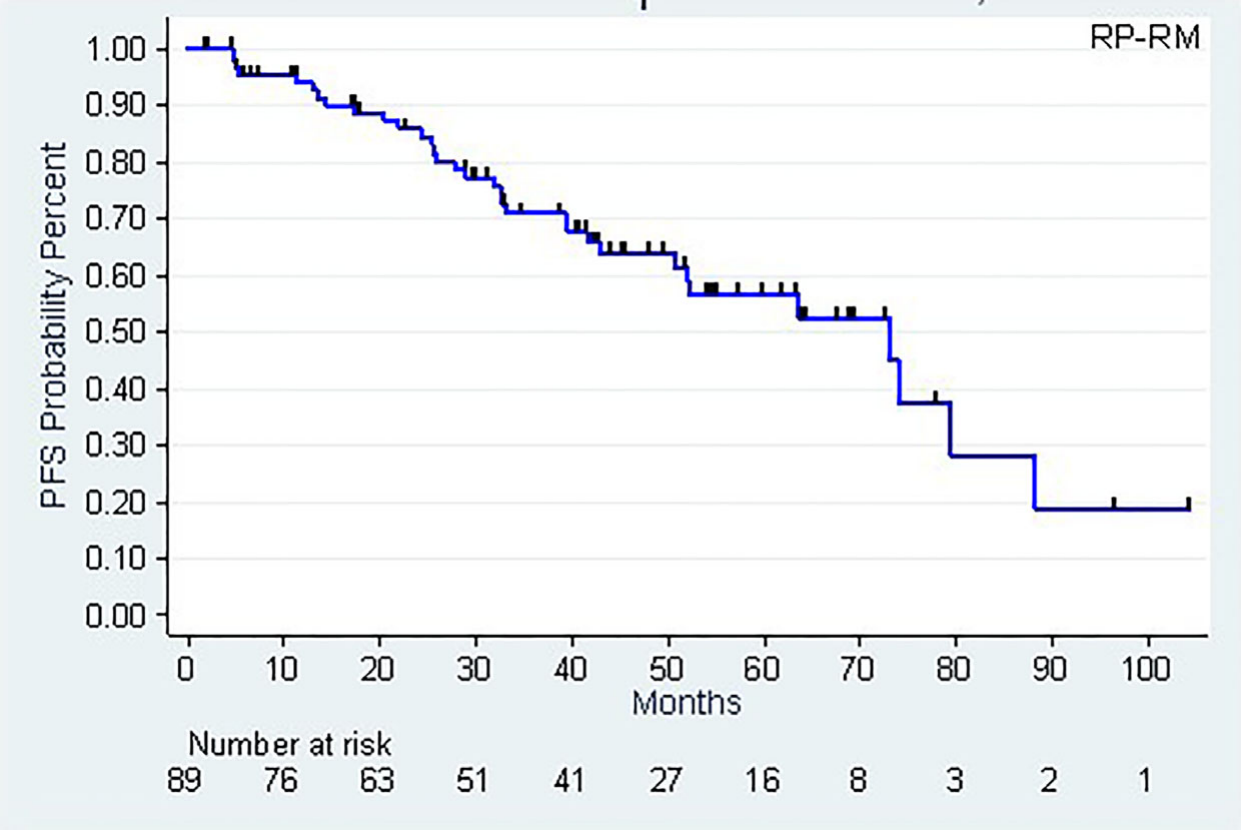
Biochemical progression-free survival (PSA nadir + 0.5 ng/mL).

The median biochemical progression-free survival (CI95%) were 60.1 months (39.3 – 73.0) and 73.0 months (50.7 – 88.2), according to PSA > 0.2 ng/mL (Definition 1) or post-RT PSA nadir + 0.5 ng/mL (Definition 2), respectively. The 5-year biochemical progression-free survival rates (CI95%) were, respectively, 50.8% (36.7 – 63.3) (Definition 1) and 56.6% (42.7 – 68.2) (Definition 2) (**Table 4**, **Figure 3**).

**Table 4:** Biochemical progression-free survival.

Characteristics	According to PSA > 0.2 (Def. 1)		According to post-RT PSA nadir+ 0.5 (Def 2)	
**Survived with no recurrence**				
Number of progressions or deaths	37^(1)^		33^(2)^	
Median (months) (CI95%)	60.1 months	(39.3 – 73.0)	73.0 months	(50.7 – 88.2)
5-year rate (%) (CI95%)	50.8%	(36.7– 63.3)	56.6%	(42.7 – 68.2)
8-year rate (%) (CI95%)	16.4%	(3.3 – 38.4)	18.8%	(3.8 – 42.4)

^(1)^32 biochemical progressions, PSA > 0.2 & 5 deaths with no prior biochemical progression.

^(2)^28 biochemical progressions, post-RT PSA nadir + 0.5 & 5 deaths with no prior biochemical progression.

The average time between the radiotherapy and the biochemical progression (Definition 2) was 2.8 years (SD = 1.9).

Metastatic recurrence occurred in 11.2% of the patients, with 7% of them presenting with bone metastasis.

We performed an analysis of the impact of the prognostic and therapeutic factors (tumoral stage, post-op Gleason score, resection margins, pre-RT PSA level, PSA kinetics, size of the macroscopic recurrence, boost to the macroscopic recurrence, hormone therapy, etc.) on the biochemical progression-free survival rate. None of these factors was significantly associated with biochemical progression-free survival in univariate analysis. Furthermore, we did not observe any significant heterogeneity of Boost effect in terms of biochemical progression-free survival according to the status of hormonotherapy.

With regard to the tolerance of the radiotherapy, 62% of the patients had acute urinary toxicity, of grade 1 in 47% of the cases. 53% of the patients developed delayed urinary toxicity of which 40% were grade 1. The side effects were mostly irritative signs of the lower urinary tract (pollakiuria, urgency). Late hematuria occurred in 4 patients in the Boost group and was grade 1 and 2.

20% and 8%, respectively, developed acute and delayed digestive toxicity. Escalating the radiotherapy dose to the macroscopic nodule in the prostate bed did not seem to increase either the risk or the severity of the acute or delayed urinary and digestive toxicity (p > 0.5). 

## Discussion

Our work is a descriptive retrospective study of a series of 89 patients with a macroscopic recurrence in the prostate bed and who underwent radiation therapy at the Oscar Lambret Centre. It is one of the series with the largest number of participants published to date (10–12) (**Table 5**).

**Table 5:** Comparative table of the results of the main retrospective studies.

	Number of pts	RT dose (Gy)	Median PSA (ng/mL)	Median follow-up	bPFS %	mPFS %	OS %
**Shelan et al.** (**10**)	69	PB: 64–66 Boost: 72-74 (2 Gy/Fr)	2.7 (0.9–6.5)	38 months	3 years 58% 5 years: 44%	3 years 91% 5 years: 76%	ND
**A.Bruni et al.** (**11**)	105	> 70: 58 pts, 66–70: 43 pts, < 66: 4 pts.	29 pts: PSA < 1.0 50 pts: 1.1<PSA< 5, 25 pts: > 5	52 months	5 years: 69.7% 10 years 57.7%	5 years: 86.1% 10 years 73.3%	5 years: 85.5% 10 years 76.1%
**Zilli et al.** (**12**)	171, of which 131 LR	PB: 64 (1.8- 2Gy/FR) Boost: 74 (2 Gy/Fr)	0.75 (0.1-15.6)	36 months	3 years 64.2% Boost (n = 131) 68.4 No boost (n = 40) 49.7 p.: 0.251 5 years: 45.6%	3 years 93.4 ± 3.3%, 5 years: 85.2 ± 3.2%	3 years 100% 5 years: 92.5%,
**Our series**	89	Median - (Range) 70 (60; 74) Mean – SD 68.8 2.5	0.4 (0.19 – 8)	53.7 months	5 years: 50.8% 8 years 16.4%	5 years: 76.6% 8 years 57.2%	5 years: 90.2% 8 years 69.8%

pts, patients; RT, radiotherapy; LR, Local Recurrence; bPFS, biochemical-progression-free survival; mPFS, metastatic progression-free survival; OS, overall survival; ND, not documented; PB, Prostatectomy bed; Boost, supplementary dose.

Salvage radiation is the only potentially curative therapy for biological progression after radical prostatectomy. It is associated with an improved biochemical progression-free survival rate, an improved metastatic progression-free survival rate, and an improved survival rate overall (13). Several studies have shown that the efficiency of salvage RT is highly dependent on the prostate-specific antigen (PSA) level prior to radiotherapy (14).

Magnetic resonance imaging appears to be one of the best diagnostic tools for detecting local recurrence when the PSA level is below < 1 ng/mL (8). Thus, radiation oncologists are increasingly faced with occurrences of macroscopic recurrences in the prostate bed. In our series, 58% of the patents with a macroscopic nodule visible under MRI have a PSA less than or equal to 0.5 ng/mL. Choline PET is associated with improved sensitivity and specificity on lymph node recurrences (15). PSMA PET CTs are more sensitive and can be suggested for patients whose PSA level is less than 0.5 ng/mL (16, 17).

The PSA level prior to salvage radiotherapy is a prognostic factor in the radiotherapy response. In a meta-analysis by Ohri et al, a 1 ng/mL increase in the pre-RT PSA reduces 5-year biochemical progression-free survival by 18.3% (CI of 95%: 10.4%-26.3%) (18). In our series, we did not find any statistically significant correlation between the pre-RT PSA level and the biochemical progression-free survival rate, though this may have been due to the hormone treatment prescribed for about half of the patients.

The optimal dose indicated to treat microscopic disease in the prostate bed is 64-66 Gy (19, 20), which may be insufficient if macroscopic disease is found in the bed. Increasing the dose in this category of patients may be necessary to get therapeutic results comparable to those of patients with no macroscopic disease.

In our analysis, the 5-year biochemical progression-free survival rate was 50.8% ((CI95%): 36.7-63.3); the 5-year metastatic progression-free survival rate was 76.6% ((CI95%): 62.7-85.9). Our results are very similar to those of the retrospective study by Shelan et al. (10); all of the patients in that study were treated uniformly with image-guided dose-escalated RT to the macroscopic recurrence: 3 to 5-year biochemical progression-free survival was 58% and 44%, respectively, and 3 to 5-year clinical survival was 91% and 76%.

In our analysis, we did not find any statistically significant difference between radiotherapy with or without boost, with regard to biochemical progression-free survival; nor did they in the retrospective study by A. Bruni et al. (11) in which no statistical advantage was found in the group receiving the increased dose (>70 Gy) with regard to OS or to mPFS. In another study by Zilli et al. (12), there is no significant difference in 3-year biochemical-progression-free survival between standard prostate bed therapy targeting a microscopic disease and boosted treatment if a nodule is identified by MRI (74 Gy: 68.4 months ± 4.6/64 Gy: 49.7 months ± 10.0).

These various results raise the question of whether there is any interest in escalating the dose to the macroscopic nodule; however, in these various studies, as in ours, the hormone therapy could have masked a potential benefit. In our study, the increase in the dose to the macroscopic nodule was also limited and might explain these negative results.

With regard to post-radiotherapy toxicity, a prospective study that assesses the escalated dose of post-operation radiotherapy (64Gy vs.70Gy), in the absence of any detectable local recurrence, is the SAKK 09/10 study. This study showed low, grade 2 and grade 3 U and GI toxicity rates with minor impact on urinary quality of life (21). In the Ohri series (18), late GI and GU toxicity increased with salvage radiotherapy dose by 1.2% per Gy (p=0.012) and 0.7% per Gy (p=0.010), respectively. In our series, escalating the radiotherapy dose to the macroscopic nodule on the prostate bed did not significantly increase the risk and severity of acute and delayed post-radiation toxicity.

Due to the lack of standard management of this category of patients, a prospective study must be undertaken to better define the place of dose escalation, radiotherapy regimens, as well as that of hormone therapy, and thus to standardize care. In this regard, a prospective study, “The MAPS Trial” (NCT01411345) is underway. It assesses dose escalation in light of the recurrence detected in post-prostatectomy MRI (68 Gy, in 2 Gy/fraction to the prostatectomy site and concomitant boost of 2.25 Gy/fraction, for a total dose of 76.5 Gy).

## Conclusion

Salvage radiotherapy has its place in the management of patients with biochemical progression with local recurrence in the prostate bed, with an acceptable toxicity profile. The interest of the boost is to be evaluated in prospective trials.

## Data Availability Statement

The raw data supporting the conclusions of this article will be made available by the authors, without undue reservation.

## Ethics Statement

Ethical review and approval was not required for the study on human participants in accordance with the local legislation and institutional requirements. Written informed consent for participation was not required for this study in accordance with the national legislation and the institutional requirements.

## Author Contributions

DP and HZ designed the study. HZ performed the data collection. DP, BV, BB, TL, XM and EL performed the patients’ recruitment and the follow-up. JW performed the statistical analysis. HZ wrote the original draft. DP supervised the project. All authors helped revising and editing the manuscript.

## Conflict of Interest

The authors declare that the research was conducted in the absence of any commercial or financial relationships that could be construed as a potential conflict of interest.
